# Cat eye syndrome caused by 22q11.1q11.21 duplication: case report in a Chinese family

**DOI:** 10.1186/s13039-023-00660-2

**Published:** 2023-10-25

**Authors:** Yanan Wang, Pai Zhang, Yuqiong Chai, Weiwei Zang

**Affiliations:** Department of Genetics and Prenatal Diagnosis, Luoyang Maternal and Child Health Hospital, No. 206 Tongqu Road, Luolong District, Luoyang, 471000 Henan Province China

**Keywords:** Cat eye syndrome, Chromosome 22, 22q11.1q11.21 duplication, Supernumerary small marker chromosomes, CNV-seq

## Abstract

**Purpose:**

This paper presents a report on two uncommon instances of cat eye syndrome in a Chinese family.

**Case presentation:**

The proband, a 23-year-old female, exhibited a diminutive cornea and complete blindness in her right eye, and the uncorrected distance visual acuity of her left eye was 0.7 LogMAR. Peripheral blood chromosome karyotyping reveal a karyotype of 47, XX, + mar. Subsequent analysis of chromosome copy number variation unveiled a 1.5 Mb duplication in the 22q11.1q11.21 region of the proband. The proband's mother,aged 49, displayed small eyes, wide-set eyes, downward slanting eyelids, and congenital heart disease. Chromosome copy number variation analysis also showed a 1.55 Mb duplication in the 22q11.1q11.21 region of chromosome 22 in the proband's mother. Ultimately, both members of this family were diagnosed with cat eye syndrome.

**Conclusion:**

Cat eye syndrome is a rare genetic disorder that greatly affects patients' lives and requires personalized treatment. This study provides new evidence for a better understanding of the diagnosis of cat eye syndrome and emphasizes the importance of genetic counseling and supervision.

## Introduction

Cat eye syndrome (CES) is a rare chromosomal abnormality syndrome, with an incidence rate ranging from approximately 1 in 50,000 to 1 in 150,000 [1]. This disease is mainly caused by supernumerary small marker chromosomes (sSMCs) formed by partial trisomy or tetrasomy of the short arm and long arm of chromosome 22 [2]. Due to the vertical iris defect, this syndrome is named "cat eye". The main symptoms of Cat eye syndrome include eye abnormalities and other congenital malformations, which can exhibit variability in expression due to individual differences [3]. Eye abnormalities: Patients often present with microphthalmia on one side of the eye and abnormal eye color, usually blue or hazel. Some patients may also have other eye abnormalities, such as missing irises, iris malformations, cataracts, and vitreous clouding, which can affect vision, in severe cases, lead to complete loss of vision [4]. Downslanting palpebral fissures, cleft palate, congenital heart, urinary tract defects, skeletal anomalies and mental retardation are also common [5]. Other midline defects such as spina bifida were also reported [6, 7]. In addition, renal and gastrointestinal malformations have been reported in severe cases. Although cognition is usually normal, 30% of patients have an intellectual deficit [8]. In conclusion, CES has a variable clinical spectrum, from patients with minor dysmorphias to patients with severe malformations.

Currently, the deletion or duplication of genes in the 22q11.1-q11.21 region has been found to be one of the main causes of Cat eye syndrome [9]. This region contains multiple genes, some of which have been identified to be associated with the occurrence of Cat Eye Syndrome. Gene variations can affect important physiological processes such as the eyes, heart, immune system, intelligence, and others. Therefore, studying and understanding this region is important for the treatment and prevention of Cat Eye Syndrome.

In this article, we diagnosed two rare Cat eye syndrome patients in a Chinese family through cytogenetic karyotype analysis combined with CNV-seq detection technology. The findings of this study offer significant insights for future investigations into the etiology and diagnosis of Cat Eye Syndrome, while also emphasizing the significance of recognizing familial clustering in genetic diseases.

## Case presentation

Proband II-1, a 23-year-old female, sought genetic counseling at our hospital’s clinic during her pregnancy. A thorough assessment was conducted, including a comprehensive physical examination, imaging studies, laboratory tests, and a review of her medical and family history. To prepare genomic DNA, 5 mL of peripheral venous blood was collected from both the proband and her mother, I-2.

The proband described herself as having a smaller than average cornea in her right eye, total absence of vision in her right eye, and the uncorrected distance visual acuity of her left eye was 0.7 LogMAR. Physical examination revealed a corneal diameter of approximately 8 mm in the right eye, iris defect, inner protrusion of the eyebrow, wide interorbital distance, downward slanting of the upper eyelid, small jaw, and dull facial expression. She also delayed growth in childhood. No other abnormalities were found in other systems.

**Fig. 1:**
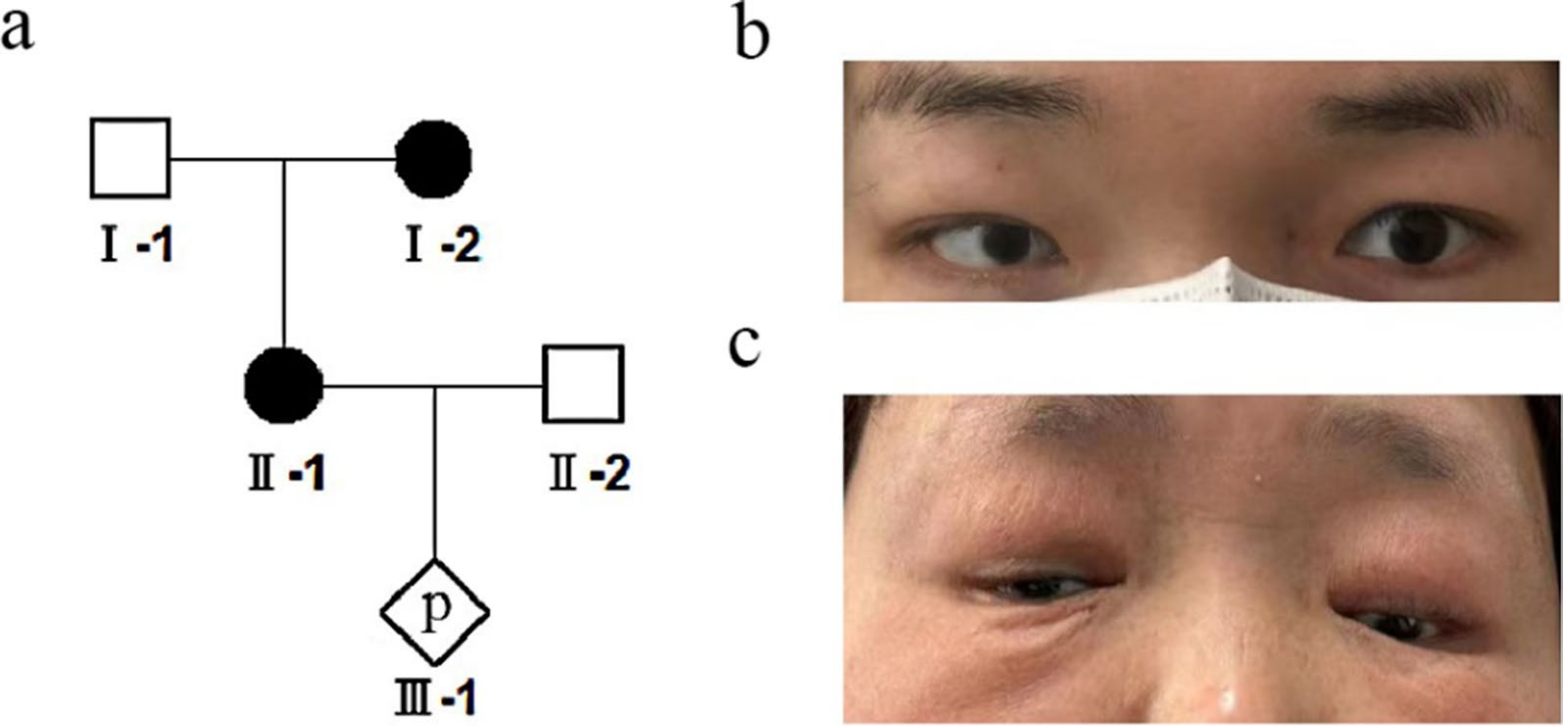
**a** The pedigree of the proband. II-1 is the proband and I-2 is the proband's mother. **b** The proband's phenotype is presented, with a corneal diameter of approximately 8 mm in the right eye, iris defect, wide intercanthal distance, prominent inner eyebrows, small jaw, and a blank expression. **c** The phenotype of the proband's mother is shown, with iris defects in both eyes, poor vision, small eyes, and downward slanting of the upper eyelids

The proband's mother had similar symptoms: vitreous opacity, small eyes, wide interorbital distance, and downward slanting of the upper eyelid. In addition, the patient's mother also had congenital heart disease. The mother was 25 years old during pregnancy, described no medication history during pregnancy, no X-ray exposure or toxic exposure history, non-consanguineous marriage, and no family history of genetic diseases. The proband's father and husband had normal physical examinations and no abnormal symptoms. The family lineage of the proband is shown in Fig. 1a.The proband's and her mother's phenotypes are shown in Fig. 1b and c.

**Fig. 2:**
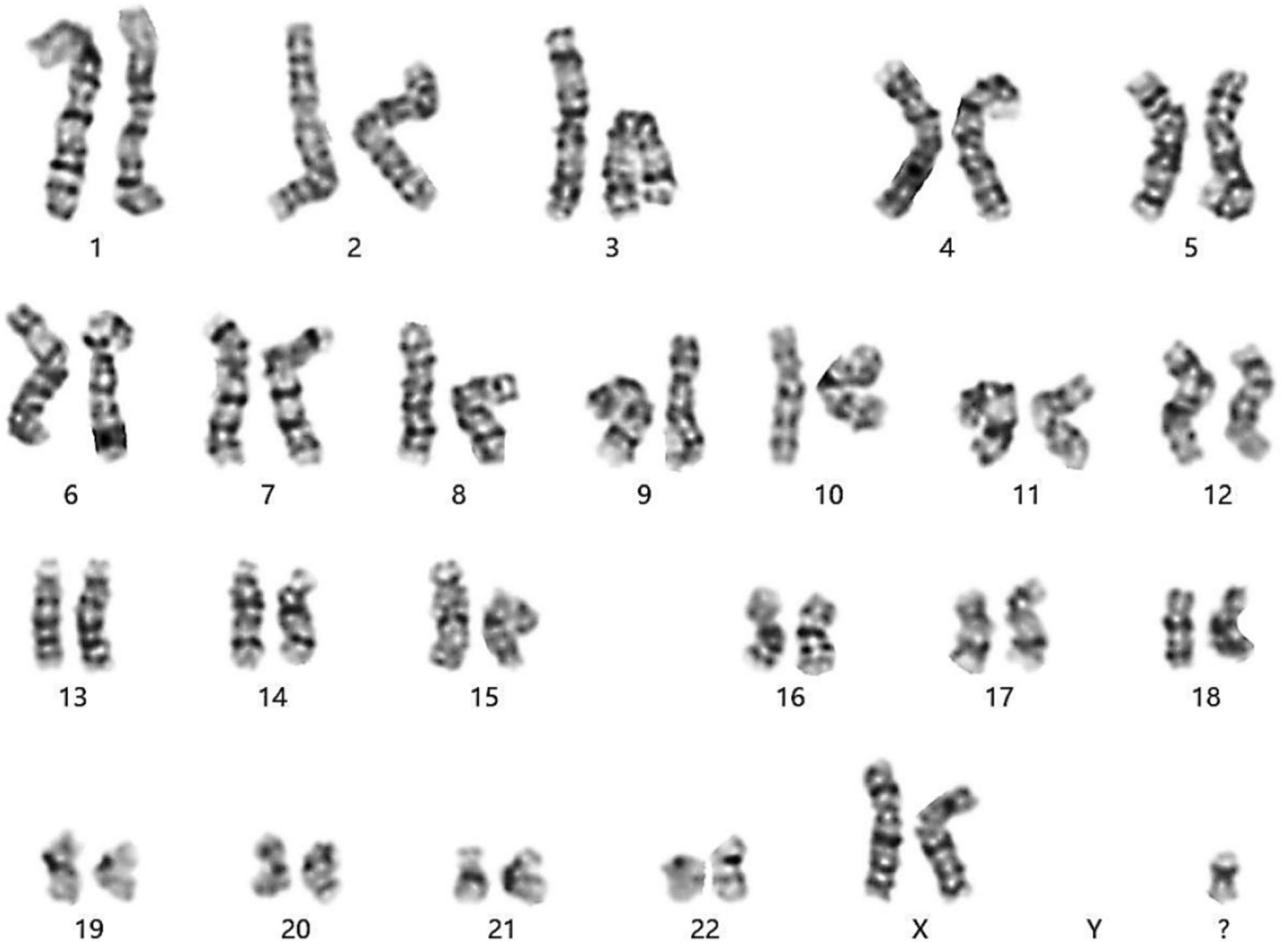
Karyotype analysis of the proband (with more than 320 bands) by G-banding method

We collected 2 mL of peripheral venous blood from the proband using aseptic technique, and performed in vitro cell culture, harvest, fixation, karyotype preparation, and G-banding, under a light microscope for chromosome karyotype analysis. Chromosome karyotypes were described according to the International System for Human Cytogenetic Nomenclature (ISCN) 2020 [10]. In the peripheral blood chromosome karyotype test, we observed that the proband's karyotype was 47,XX, + mar (Fig. 2). + mar refers to unidentified extra marker chromosome, supernumerary small marker chromosomes (sSMC), which can only be distinguished morphologically by conventional G-banding karyotype analysis, but its origin and genomic composition cannot be clearly diagnosed. This result suggests that the proband may have chromosomal abnormalities and further testing and confirmation are required.

**Fig. 3:**
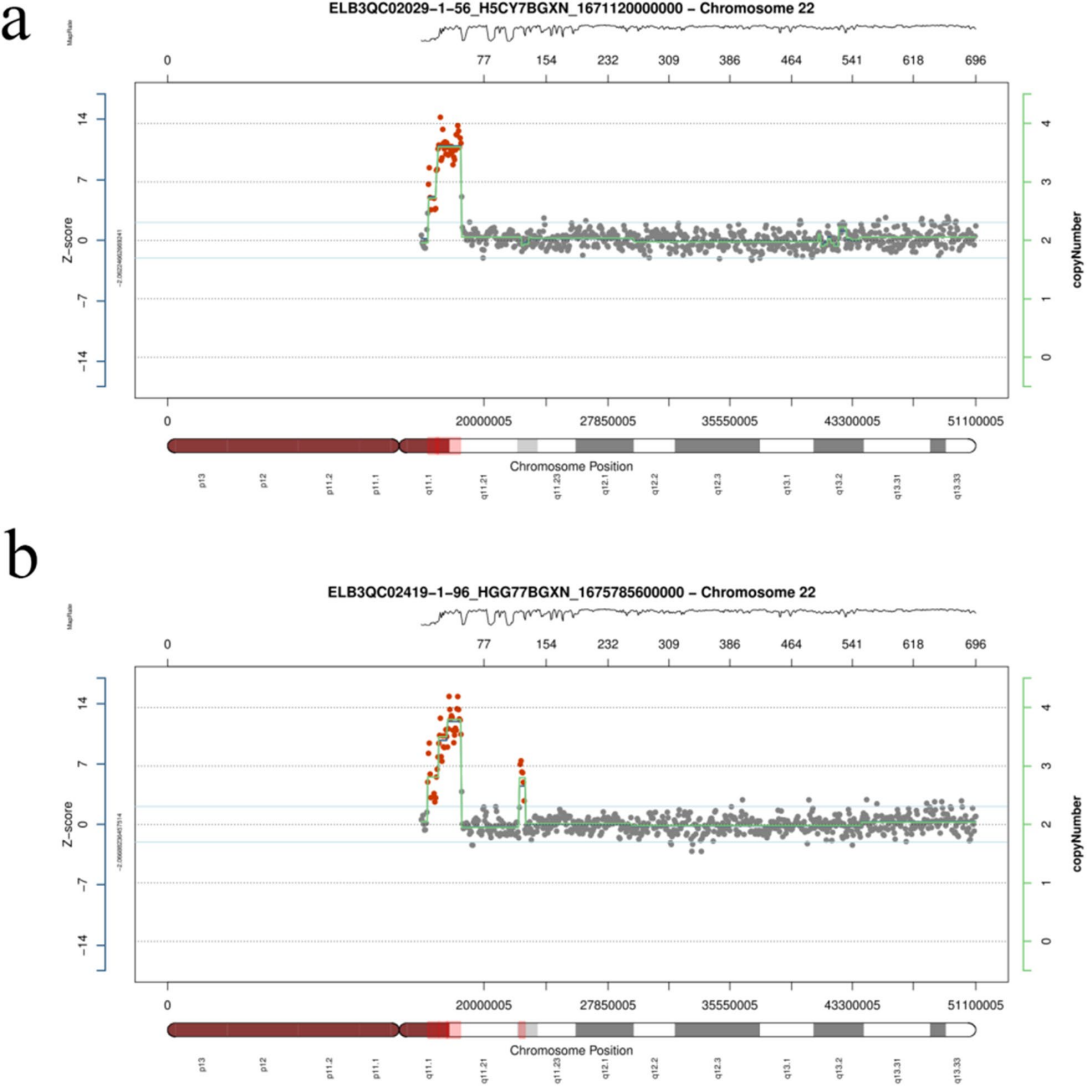
**a** The CNV-seq detection results for the proband. **b** The CNV-seq detection results for the proband's mother. Both show copy number variations in the 22q11.1q11.21 region of chromosome 22

To determine the origin of the supernumerary small marker chromosomes (sSMCs) found in the proband, we performed copy number variation sequencing (CNV-seq) analysis. The specific steps were as follows: (1) DNA was extracted from the peripheral blood sample of the proband. (2) The DNA was fragmented according to the instructions of the Chromosome Aneuploidy Test Kit (Reversible Terminator Sequencing) from Annoroad (Beijing). (3) The fragmented DNA was subjected to end repair, adaptor ligation, tagging, and PCR amplification to construct a sample library, which was then quantified by fluorescent PCR and subjected to sequencing. (4) Data analysis and result interpretation were performed by comparing the obtained DNA sequences with the human reference genome, using the CNV analysis software from Annoroad to determine the presence of copy number variations. Furthermore, we searched the DGV (Database of Genomic Variants), Decipher, OMIM (Online Mendelian Inheritance in Man), ClinGen (Clinical Genome Resource), Clinvar databases, and reviewed relevant literature (PubMed), and referred to the guidelines of the American College of Medical Genetics and Genomics to determine the nature and clinical significance of the copy number variations. Results analysis: By comparing with the human reference genome GRCh37/hg19, a copy number variation (CNV) of approximately 1.5 Mb in size was detected in the 22q11.1-q11.21 region of the sample, with the specific location being chr22:g.17100001_18650000dup (Fig. 3a). There were no reports of polymorphic cases found in the DGV database. The duplicated region completely overlapped with the 22q11.21 recurrent (Cat eye syndrome) region (includes CECR2) (chr22:17,392,953–18,591,860), and the current ClinGen database three-star dosage sensitivity score was 3, indicating clarify pathogenic variants. Therefore, according to the 2019 ClinGen/ACMG CNV interpretation guidelines, this CNV is classified as a pathogenic variant. To determine if the proband's mother had the same pathogenic variant, CNV-seq testing was also performed. The results showed that the proband's mother also had a 1.55 Mb duplication in the 22q11.1q11.21 region of chromosome 22 (chr22:g.17150001_18650000dup) (Fig. 3b).

**Fig. 4:**
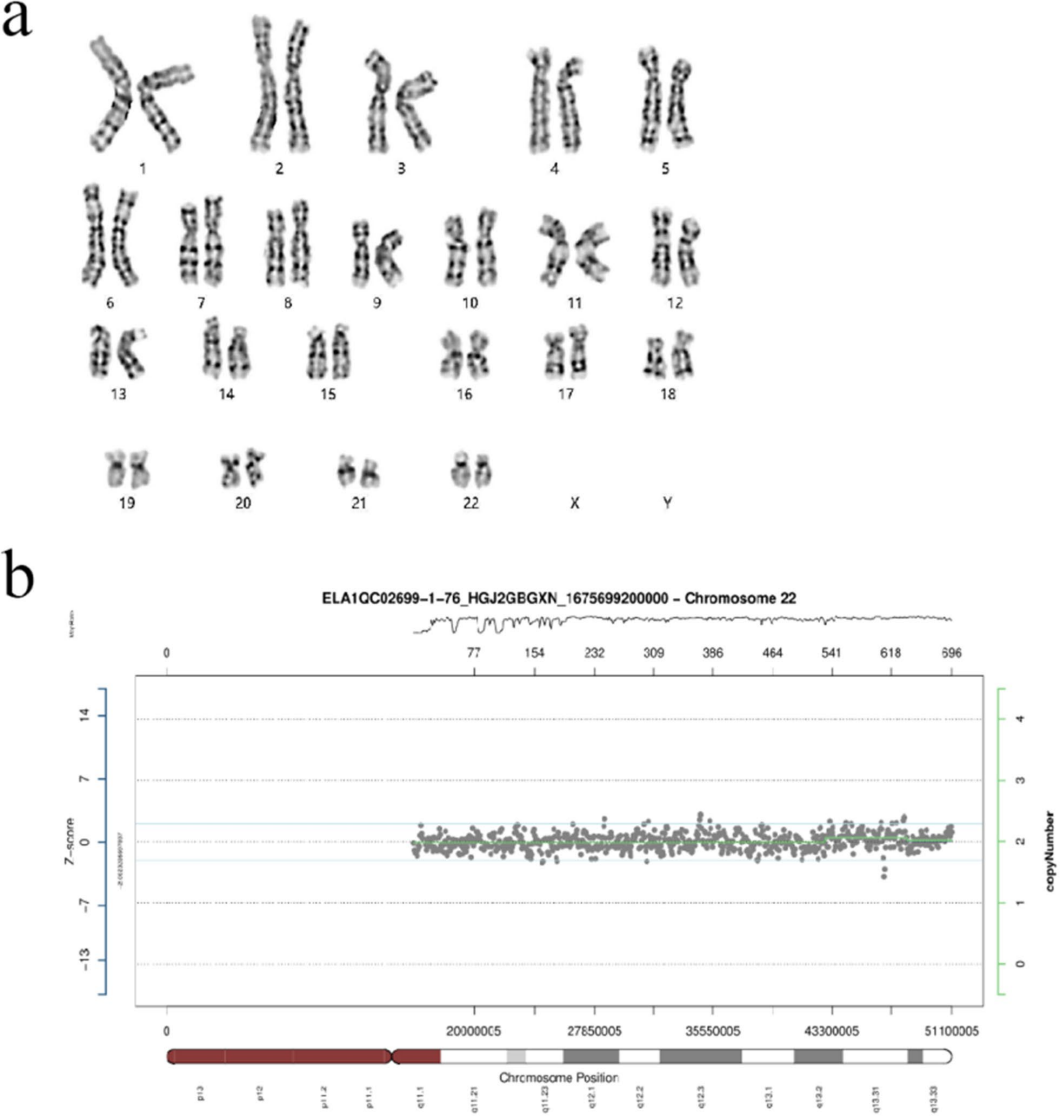
**a** Karyotype analysis of the proband's fetus (with more than 320 bands) by G-banding method. The karyotype is normal. **b** The CNV-seq detection results for the proband's fetus. This result shows no exceptions

This result further confirms the association between gene deletions or duplications in the 22q11.1q11.21 region and the occurrence of Cat Eye Syndrome, providing a clear diagnosis for the patient and her family. Based on our testing results, the patient needs further medical evaluation and treatment. We also need to further study and understand the impact mechanism of this CNV in order to provide better treatment and rehabilitation plans. Fortunately, the proband is currently 21 weeks pregnant, and we performed amniocentesis to extract 30 ml of amniotic fluid for chromosomal karyotyping and CNV-seq testing, and no abnormalities have been detected (Fig. 4a and b). Based on this case, we call for broader genetic testing and genetic counseling to better diagnose and manage this rare hereditary disease.

## Discussion

Cat eye syndrome is a rare genetic disorder involving the proximal long arm of chromosome 22. It has been shown that partial tetrasomy 22q11.1q11.21 is a key region in cat eye syndrome [11]. 22q11 fragment is a susceptible region for chromosomal rearrangements, where a large number of tandem repeats (TTAGGGAGG) are present, and during meiosis, U-shaped exchanges of homologous chromosomal monomers constitute the long-arm partial tetrasomy karyotype [12, 13]. One of the features of cat eye syndrome is the variable clinical presentation, and initially, iris defects, preauricular redundancy, and anal atresia were thought to be typical features of this syndrome. However, of the cases ever reported, there may be no iris defect in 40–50%; those with both iris abnormalities and anal atresia are only a minority [14]. Cat eye is known as coloboma, i.e. failure to close a fissure in the lower part of the eye during early development, which gives the eye a keyhole appearance. It may involve the iris, choroid and the retina. If only the iris is affected, vision is not affected but if the choroid and retina are affected as well, vision will be affected and the patient may develop blindness. They may present with other eye abnormalities including strabismus, cataract, aniridia or microphthalmia. Fewer cases of this syndrome have been reported. The two patients with cat eye syndrome that we described did not present with the typical clinical triad. The patients only showed iris defects, small eyes, wide eye spacing, and downward-sloping upper eyelids, but no deformities of the ears or anus were found. The iris defect was one of the important clues. Therefore, in the absence of the classical triad, genetic studies are crucial for the final diagnosis.

The limitations of karyotyping do not allow for accurate analysis of marker chromosomes, which in turn provides strong evidence for genetic counseling in CES by determining the origin of marker chromosomes by CNV-seq techniques in combination with clinical phenotypes. The critical region for CES is currently estimated to be about 2.1 Mb and to contain at least 14 RefSeq genes. Gain of this region may cause ocular coloboma, preauricular, anorectal, urogenital and congenital heart malformations. The region 22q11.1q11.21 of our reported proband has a 1.55 Mb repeat, which is classified as a pathogenic variant of the CNV. This fragment contains 15 protein-coding genes or gene fragments including *TUBA8*, *PEX26*, *USP18*,etc. Six of these OMIM morbid genes. Among these genes, amplification of the genes *CECR2*, *SLC25A18* and *ATP6V1E1*, mapping within the critical region for CES, may be responsible for iris coloboma,congenital heart disease, craniofacial anomalies in patients with CES. In previous studies [15], patients with cat eye syndrome often had de novo variants, but in the cases we report, the pathogenic variants in the proband were derived from her mothers, but her phenotypes were not as severe as those of her mother. Some cases of CES are not secondary to atopy but involve familial translocations of chromosome 22, in which case the recurrence rate may be higher, making genetic counseling crucial for such families.

At present, there exists no definitive curative intervention for cat eye syndrome; however, the mitigation of symptoms can be achieved through surgical procedures, congenital heart disease treatment, educational initiatives, and supportive measures. Given its hereditary nature, individuals with a documented familial background of cat eye syndrome should prioritize genetic counseling to comprehend associated risks and facilitate preventive measures and effective management.

## Conclusion

This study reports a case of a 23-year-old pregnant woman with congenital eye disorders, including the micro cornea and strabismus. Her mother also has similar symptoms and has congenital heart disease. Through chromosomal karyotyping and CNV-seq testing, we identified a small, unidentified extra marker chromosome (sSMC) in the proband, located in the 22q11.1q11.21 region, with a size of approximately 1.5 Mb. Our results suggest that this sSMC may be related to the proband's congenital eye disorders. Some previous reports mention that cases with mild manifestations may be due to a state of mosaicism, the case described in our report suggests that being mother and daughter affected the cytogenetic alteration is not in a constitutional state, which is a new finding that proposes that the spectrum of clinical manifestations is independent of a state of mosaicism. The findings of this study provide important information for the diagnosis and research of cat eye syndrome, as well as new clues for the genetic mechanism and pathogenic genes of this disease. Further research is expected to elucidate the pathophysiological mechanisms and clinical features of this disease, which can help improve diagnostic accuracy and disease management.

## Data Availability

Not applicable.
